# *TPL2/COT/MAP3K8 (TPL2)* Activation Promotes Androgen Depletion-Independent (ADI) Prostate Cancer Growth

**DOI:** 10.1371/journal.pone.0016205

**Published:** 2011-01-18

**Authors:** Joseph H. Jeong, Ayesha Bhatia, Zsolt Toth, Soohwan Oh, Kyung-Soo Inn, Chun-Peng Liao, Pradip Roy-Burman, Jonathan Melamed, Gerhard A. Coetzee, Jae U. Jung

**Affiliations:** 1 Department of Molecular Microbiology and Immunology, USC Norris Comprehensive Cancer Center, Keck School of Medicine, University of Southern California, Los Angeles, California, United States of America; 2 Department of Pathology, Keck School of Medicine, University of Southern California, Los Angeles, California, United States of America; 3 Department of Pathology, NYU Prostate Cancer Tissue Resource, New York University, New York, New York, United States of America; 4 Department of Urology and Preventive Medicine, USC Norris Comprehensive Cancer Center, Keck School of Medicine, University of Southern California, Los Angeles, California, United States of America; Institut de Génomique Fonctionnelle de Lyon, France

## Abstract

**Background:**

Despite its initial positive response to hormone ablation therapy, prostate cancers invariably recur in more aggressive, treatment resistant forms. The lack of our understanding of underlying genetic alterations for the transition from androgen-dependent (AD) to ADI prostate cancer growth hampers our ability to develop target-driven therapeutic strategies for the efficient treatment of ADI prostate cancer.

**Methodology/Principal Findings:**

By screening a library of activated human kinases, we have identified *TPL2*, encoding a serine/threonine kinase, as driving ADI prostate cancer growth. TPL2 activation by over-expressing either wild-type or a constitutively activated form of TPL2 induced ADI growth, whereas the suppression of *TPL2* expression and its kinase activity in ADI prostate cancer cells inhibited cell proliferation under androgen-depleted conditions. Most importantly, *TPL2* is upregulated in ADI prostate cancers of both the *Pten* deletion mouse model and the clinical prostate cancer specimens.

**Conclusions/Significance:**

Together these data suggest that *TPL2* kinase plays a critical role in the promotion of ADI prostate cancer progression. Furthermore, the suppression of TPL2 diminishes ADI prostate cancer growth and a high frequency of TPL2 overexpression in human ADI prostate cancer samples validates TPL2 as a target for the treatment of this deadly disease.

## Introduction

Prostate cancer affects 1 in 6 American men, with more than 2 million currently living with the disease. Surgery is effective, with nearly 100% of the patients remaining cancer-free for several years if the disease is detected early and the cancer cells are confined to the prostate [1]. However, once the cancer spreads beyond the prostate gland, the outcome is nearly always fatal. Since prostate cancer cells require testosterone to fuel their growth and survival, androgen-deprivation therapy has been designed to halt cancer growth by either stopping the production of testosterone or preventing the hormone from acting on prostate cancer cells [2]. Despite the initial positive response to hormone therapy, prostate cancer cells invariably recur in an aggressive, androgen depletion-independent (ADI) form. While genetic alterations associated with the initiation and progression of prostate cancer have been intensively studied [3], [4], [5], [6], [7], those underlying the transition from androgen-dependent (AD) to ADI prostate cancer growth are relatively less well understood. This lack of understanding hampers our ability to develop target-driven therapeutic strategies for the efficient treatment of ADI prostate cancer.

In the presence of androgen, the androgen receptor (AR) undergoes phosphorylation, dimerization, and translocation into the nucleus, wherein it binds to androgen response elements (ARE) sites, resulting in the transcriptional activation of target genes [8]. However, compelling data, including RNAi knockdown and the introduction of antagonists of the androgen receptor (AR), indicate that AR is still necessary for ADI prostate cancer growth [9], [10], [11]. Therefore, under androgen-depleted conditions, ADI prostate cancer cells appear to develop intracellular strategies that activate the AR signaling pathway. Many underlying mechanisms have been proposed: increased *AR* expression through *AR* gene amplification, increased *AR* sensitivity through enhanced AR stability and nuclear localization, broadened AR ligand specificity through mutations in its ligand-binding domain, and increased AR activity through post-translational modifications [12], [13], [14], [15], [16], [17], [18]. On the other hand, the activation of other signal transduction pathways, such as BCL-2 activation, may also bypass the requisite of AR activation for the proliferation and survival of prostate cancer cells [19]. Finally, a preexisting subpopulation of ADI prostate cancer cells with progenitor/stem cells traits may become dominant under androgen-depleted conditions [20].

Many studies reveal that the activation of the RAS/RAF/MEK/ERK pathway may be correlated with ADI prostate cancer growth [5], [13], [21], [22]. Recently, a genetically-engineered mouse (GEM) model, in which the expression of a potent activator of RAS-MAPK signaling, B-RAF^E600^, is targeted to the prostate epithelium using a tet-inducible system, developed invasive adenocarcinoma, and this further progressed to indolent ADI lesions after castration [23]. However, counter-intuitively, activating mutations in the RAS/RAF/MEK/ERK pathway are infrequent in human prostate cancers, although autocrine and paracrine growth factor loops appear to activate the pathway [24], [25]. Importantly, a recent study proposed that chromosomal rearrangements of the RAF kinase pathway is an additional sources of MEK/ERK signaling activation, contributing to prostate cancer development and progression [26].

In order to identify novel kinase genes related to ADI prostate cancer growth, we screened a library of activated human kinases. Here, we report the identification of a serine/threonine kinase, *TPL2*, as driving ADI prostate cancer growth through the activation of the MEK/ERK signaling pathway and NF-κB. This provides a clue for finding additional sources of MEK/ERK pathway activation in ADI prostate cancers. Furthermore, the suppression of TPL2 diminishes ADI prostate cancer growth and a high frequency of TPL2 overexpression in human ADI prostate cancer samples validates TPL2 as a target for the treatment of this deadly disease.

## Results

### The Identification of TPL2, by Screening a Library of Activated Human Kinases

**Figure 1:**
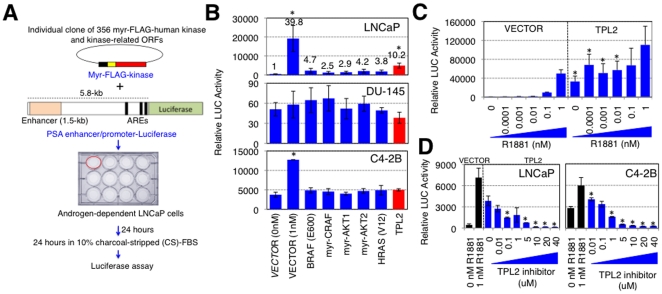
Identification of *TPL2* as a candidate gene associated with ADI prostate cancer growth. (A) Schematic of the identification of candidate genes that can induce the transcriptional activation of a *PSA* enhancer/promoter *cis*-regulatory region reporter construct under androgen-depleted conditions in androgen-dependent LNCaP cells. Myr, myristoylation sequence; ARE, androgen response elements; CS-FBS, charcoal-stripped fetal bovine serum. (B) Relative LUC activity (the *PSA* enhancer/promoter LUCIFERASE activity is normalized to the pGL3 beta-GALACTOSIDASE activity) was measured after 24 hours of incubation with 10% CS-FBS without R1881 (except for the second column with 10% CS-FBS+1 nM R1881 as a control), following co-transfection of plasmids expressing constitutively activated forms of the indicated kinase genes. The fold induction values (relative LUC activity divided by the relative basal LUC activity of the vector control) are indicated in the graph for LNCaP cells. Relative LUC activities upon *TPL2* expression are indicated in red. Statistically significant increases in LUC activities in comparison to vector control without R1881 treatment are marked with * (p<0.05). (C) Relative LUC activity with increasing concentration of R1881 in LNCaP cells transiently co-transfected with either plasmids expressing myristoylated *TPL2* or with empty vector. Statistically significant increases in LUC activity in comparison to each corresponding R1881 concentration with vector control are marked with * (p<0.05). (D) Relative LUC activity with increasing concentration of TPL2 inhibitor either in AD-LNCaP cells transiently co-transfected with plasmids expressing myristoylated *TPL2* or in untransfected AI-C4-2B cells. Relative LUC activities with/without R1881 are indicated in black as controls. Statistically significant increases in LUC activities in comparison to vector control without R1881 treatment are marked with * (p<0.05). All the experiments were performed three times with each in triplicate, and each column represents the mean ± standard deviation.

To identify novel kinase genes related to ADI prostate cancer growth, we screened a library of 354 activated human kinases composed of 98 kinase-related open reading frames (ORFs) and 256 human kinases cloned into a Gateway-compatible retroviral destination vector, pWN-MF-DEST, in which a myristoylation sequence and a FLAG-epitope tag (MF) were added to the N-termini of each introduced ORF [27]. As a readout for the screen, we used a reporter construct containing the *cis*-regulatory promoter region (5.8 kb containing an *AR* enhancer) of the prostate-specific antigen (*PSA*) gene, which is highly expressed in the normal prostate epithelium and in prostate tumors [28], [29]. The promoter/enhancer activity may be measured by a fused *Luciferase* reporter gene (Figure 1A). In order to identify possible kinase genes able to induce the transcriptional activity of the *PSA* enhancer/promoter *cis*-regulatory region under androgen-depleted conditions, each kinase construct, along with the reporter plasmid, was co-transfected into individual wells of a 12-well plate containing androgen-dependent LNCaP (AD-LNCaP) cells grown in media supplemented with R1881, a synthetic androgen (Figure 1A). Twenty-four hours after transfection, AD-LNCaP cells were incubated with the medium containing 10% charcoal-stripped fetal bovine serum (CS-FBS) without R1881. After 24 hours incubation, transcriptional activity of the *PSA* enhancer/promoter *cis*-regulatory region under androgen-depleted conditions was measured by LUCIFERASE assays. Out of 354 activated human kinase genes, the expression of *TPL2* gene in AD-LNCaP cells induced the highest level of PSA transcriptional activity in the absence of R1881 (Figure 1B upper). As controls, other oncogenes, such as *RAF* and *RAS* that have previously been known to be associated with ADI prostate cancer growth, were included [5], [22], [23], [26], [30]. Their expression indeed induced transcriptional activity of the *PSA* enhancer/promoter *cis*-regulatory region under androgen-depleted conditions to some but lesser extents than *TPL2*. However, when tested in androgen receptor (AR)-negative DU-145 prostate cancer cells, *TPL2* did not induce the ADI transcriptional activity, suggesting that AR expression is necessary for the ADI transcriptional activation of the *PSA* enhancer/promoter *cis*-regulatory region induced by *TPL2* (Figure 1B middle; please note the change in the scale of the Y-axis). On the other hand, androgen depletion-independent C4-2B (ADI-C4-2B) cells showed high endogenous levels of transcriptional activity of the *PSA* enhancer/promoter *cis*-regulatory region in the absence of R1881 (Figure 1B bottom). These data raise the possibility that preexisting genetic and epigenetic alterations in ADI-C4-2B cells contribute to the high endogenous level of ADI transcriptional activity driven by the *PSA* enhancer/promoter *cis*-regulatory region. It should be noted the induction of transcriptional activity of the *PSA* enhancer/promoter *cis*-regulatory region by *TPL2* overexpression in AD-LNCaP cells was more striking (greater than 20 fold) at low levels (0.1∼0.001 nM) of R1881, which is comparable to the physiological levels of androgen in patients who have undergone castration surgery (Figure 1C). Furthermore, ADI transcriptional activation was abrogated with a TPL2 inhibitor treatment when either with TPL2 overexpression in AD-LNCaP cells or simply with endogenous TPL2 expression in ADI-C4-2B cells (Figure 1D). To test the possibility that the high endogenous level of ADI transcriptional activity of the *PSA* enhancer/promoter *cis*-regulatory region in ADI-C4-2B cells may be due to increased TPL2 expression, we compared the expression level of *TPL2* in AD-LNCaP and ADI-C4-2B cells. Indeed, ADI-C4-2B cells showed higher level of *TPL2* expression than in AD-LNCaP cells (Figure S1A). Furthermore, in C4-2B cells, PSA enhancer/promoter activity responds to low levels of exogenous TPL2 expression, but not to high levels of TPL2 expression (Figure S1B). It is possible that high levels of TPL2 exogenous expression in C4-2B cells, which already express a higher level of endogenous TPL2 expression than LNCaP cells, result in adverse effects on PSA enhancer/promoter activity. Therefore, we have identified *TPL2* as a candidate oncogene, which may be associated with ADI prostate cancer growth.

### TPL2 Activation Induces the ADI Growth of AD Prostate Cancer Cells

**Figure 2:**
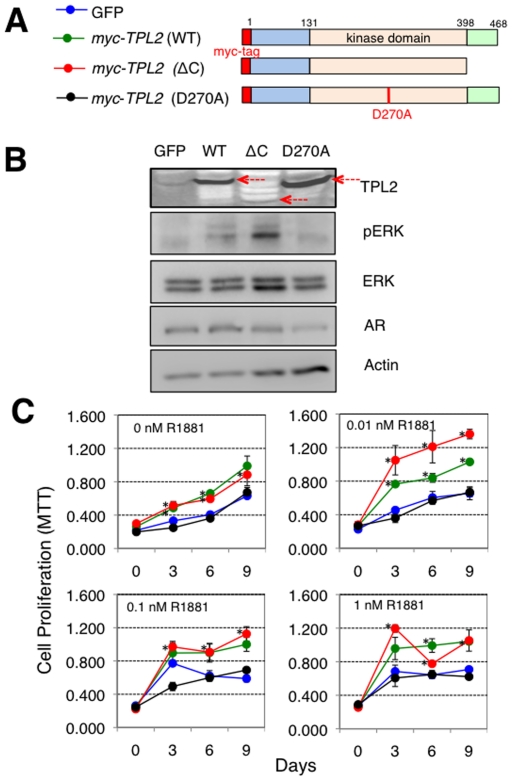
Induction of ADI prostate cancer cell growth by *TPL2* activation. (A) Schematic of established stable LNCaP cell lines overexpressing either *GFP*, a myc-tagged wild-type *TPL2* [*myc-TPL2* (WT)], a myc-tagged constitutively activated form of *TPL2* with a truncation of 70 amino acids in its C-terminus [*myc-TPL2* (ΔC)], or a myc-tagged kinase-inactive form of *TPL2* [*myc-TPL2* (D270A)] using the pBabe-puro expression vector. (B) Western blot analysis to detect the expression of the *TPL2* constructs above (as indicated with arrows in red). β-actin was used as a control for loading the same amount of proteins. (C) MTT assays to measure cell proliferation of the stable cell lines above in the absence or with low levels of R1881 (0.01 nM, 0.1 nM, and 1 nM). Statistically significant increases in cell proliferation in comparison to control cells (LNCaP-GFP) are marked with * (p<0.05).

To verify whether *TPL2* is functionally associated with ADI prostate cancer cell growth, we investigated the functional consequences of the overexpression or suppression of *TPL2* in AD or ADI prostate cancer cells, respectively. Firstly, to determine whether *TPL2* overexpression is sufficient to promote ADI prostate cancer growth, we established AD-LNCaP stable cell lines overexpressing either N-terminally myc-tagged wild-type [*myc-TPL2* (WT)], a constitutively activated form with a deletion of 70 amino acids in its C-terminus [*myc-TPL2* (ΔC)], or a kinase-inactive form of *TPL2* [*myc-TPL2* (D270A)] using the pBabe-puro vector (Figure 2A). The expression of each transduced gene was confirmed by western blot (Figure 2B). Interestingly, the expression of *myc-TPL2* (ΔC) constitutively activated mutant was lower than that of *myc-TPL2* (WT) and *myc-TPL2* D270A mutant despite having the highest level of phosphor-ERK, a downstream signaling molecule (Figure 2B). To eliminate the possibility of expression level effects, we established another set of AD-LNCaP stable cell lines that over-express either C-terminally flag-tagged wild-type (*TPL2*-wt-flag), a constitutively activated form with a deletion of 70 amino acids in C-terminus (*TPL2*-delC-flag), or a kinase-inactive form of *TPL2* (*TPL2*-inactive-flag) using the pEF-IRES-puro vector (Figure S2). This condition also showed the lower expression of *TPL2*-delC-flag than *TPL2*-wt-flag and *TPL2*-inactive-flag (Figure S2), suggesting that the expression of a constitutively activated form of *TPL2* above physiological levels may have adverse effects on the cell. However, regardless *TPL2* expression levels, overall LNCaP cells expressing either *myc-TPL2* (WT) or myc-*TPL2* (ΔC) showed accelerated cell proliferation at androgen-deprived, -low, and -rich conditions compared to LNCaP cells expressing either vector alone or myc-*TPL2* (D270A) mutant (Figure 2C). In particular, LNCaP cells expressing myc-*TPL2* (ΔC) showed statistically significant increases in cell proliferation in comparison to control cells (LNCaP-GFP) under the androgen-depleted condition and at low levels of R1881 (0.01 nM and 0.1 nM) at 3, 6, 9 days (*p-value <0.05). Similar data were obtained with stable cell lines constructed with the pEF-IRES-puro vector (Figure S2). Therefore, these data indicate that *TPL2* activation by over-expressing either wild-type or a constitutively activated form of TPL2 leads to the promotion of ADI growth of AD-LNCaP cells.

### The Suppression of TPL2 Expression or Inhibition of Its Kinase Activity in ADI Prostate Cancer Cells Results in Reduced Proliferation Phenotypes

**Figure 3:**
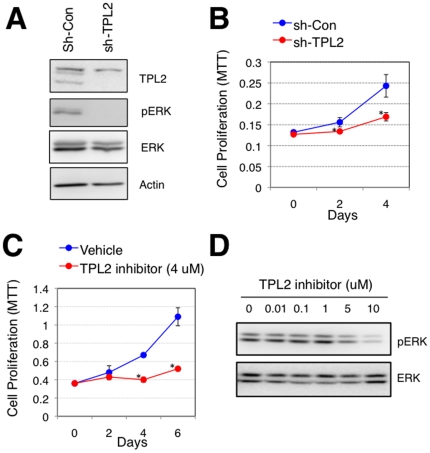
Inhibition of ADI prostate cancer cell growth by *TPL2* suppression. (A) Western blot analysis to show the amount of *TPL2* expression in the stable C4-2B cell lines expressing the indicated shRNAs using the pLKO.1-TRC lentiviral vector at the time of the following MTT assay. β-actin was used as a control for loading the same amount of proteins. (B) MTT assays to measure cell proliferation of the stable cell lines in the absence of R1881. * P<0.05. (C) MTT assays to measure cell proliferation of androgen depletion-independent C4-2B cells with the treatment of TPL2 inhibitor (4 µM). (D) The suppression of MEK/ERK pathway in ADI-C4-2B cells with increasing concentration of a TPL2 inhibitor was confirmed by western blot.

Next, to determine whether ADI prostate cancer cells are dependent on *TPL2* for their ADI cell growth, we suppressed *TPL2* expression or activity either by shRNA-mediated knockdown or by TPL2 inhibitor treatment, respectively. As shown in Figure S1, *TPL2* expression was is higher in ADI-C4-2B cells than in AD-LNCaP cells. The decreased expression of *TPL2* in ADI-C4-2B cells by shRNA-mediated knockdown using the pLKO.1-TRC vector was confirmed by western blot (Figure 3A). ADI-C4-2B cells with a decreased expression of *TPL2* showed significantly reduced proliferation rate under androgen-depleted conditions (Figure 3B). We confirmed these data by additional shRNA-mediated knockdown system using the pGIPZ lentiviral vector (Figure S3). Furthermore, the ADI cell growth of ADI-C4-2B cells was also significantly diminished upon the treatment of a TPL2 inhibitor [Figure 3C; The 50% inhibitory concentration (IC^50^) of 4.0 µM was determined as in Figure S4]. The suppression of MEK/ERK pathway in ADI-C4-2B cells with the treatment of TPL2 was confirmed by western blot (Figure 3D). Together, these data indicate that *TPL2* activation is necessary for the efficient ADI growth of ADI-C4-2B cells.

### TPL2 Activation Promotes ADI Prostate Cancer Growth in the Pten Deletion Mouse Model

**Figure 4:**
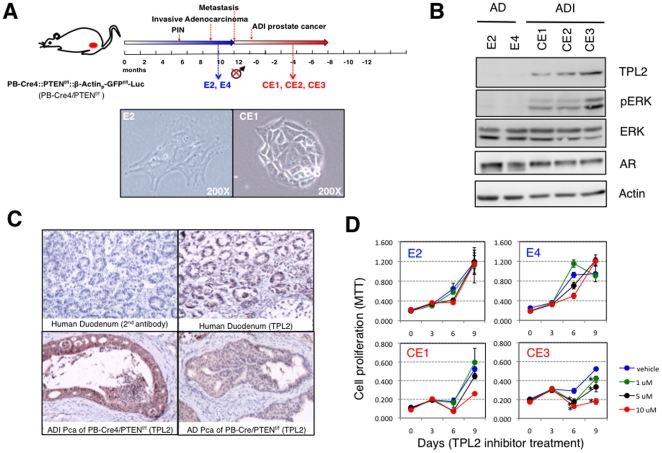
TPL2 activation in ADI prostate cancer of the *Pten* deletion mouse model. (A) Schematic of the “Pb-Cre4::Pten^f/f^::β-Actinp-GFP^f/f^-Luc” mouse model, in which *Pten* alleles are homozygously deleted and *Luciferase* is expressed in the prostate epithelium by the prostate-specific *Probasin* promoter (Pb)-driven Cre recombinase expression. These compound mice developed prostate Intraepithelial neoplasia (PIN), prostatic invasive adenocarcinoma, and metastatic prostate cancer at the indicated times. Most importantly, androgen ablation of these mice by surgical castration led to the emergence of ADI prostate cancer growth at times varying from 7 to 28 weeks post-castration in all living mice. E2 and E4 prostate cancer cell lines were established initially from primary prostate cancer cells before castration that later evolved ADI growth upon long-term culture under androgen-depleted conditions. However, CE1, CE2, and CE3 prostate cancer cell lines were established from ADI prostate cancers after castration. Photos (200X) are representative of cell morphologies of E2 and CE1 cells. (B) Western blot analysis to compare the expression levels of AR and TPL2 between E cell lines (E2 and E4) and CE cell lines (CE1, CE2, and CE3) cells. *β-actin* was used as a control for loading the same amount of proteins. AD, androgen-dependent; ADI, androgen depletion-independent; and AR, androgen receptor. (C) Immunohistochemical analysis to examine *TPL2* expression in AD and ADI prostate cancers of the *Pten* deletion mouse model. Human duodenum tissue expressing high levels of endogenous TPL2 was used as a positive control (upper right). As a negative control, the tissue was stained only with the secondary antibody (upper left). Pca, prostatic adenocarcinoma. (D) MTT assays to measure cell proliferation of E cells (E2 and E4) and CE cells (CE1 and CE3) with the treatment of different levels of TPL2 inhibitor (0, 1, 5, or 10 µM). Statistically significant decreases in cell proliferation in comparison to vehicle treatment are marked with * (p<0.05).

Previously, it had been shown that biallelic conditional Pten deletion in the prostate epithelium could induce adenocarcinoma that recapitulates most of the histopathological features of human prostate cancer [31], [32], [33]. More importantly, androgen ablation of these mice by surgical castration led to the emergence of ADI prostate cancer growth (Figure 4A) [33]. Recently, prostate cancer cell lines were established from primary prostate cancers of the *Pten* deletion model [34]. After long-term culture of these cells under androgen-depleted conditions, ADI prostate cancer-like cells designated as “E” cells evolved following an initial massive cell death of AD cells. Additionally, prostate cancer cell lines were also established from recurrent ADI prostate cancer that developed after surgical castration and named “CE” cells [34]. Unlike the E cells, these CE cell lines are androgen depletion-independent from the beginning such that there was no initial massive cell death under androgen-depleted conditions. To investigate whether *TPL2* activation is physiologically relevant to ADI tumor growth *in vivo*, we first compared the expression levels of *TPL2* between E cell lines (E2 and E4) and CE cell lines (CE1, CE2, and CE3). CE cell lines (CE1, CE2, and CE3) showed higher levels of *TPL2* expression compared to E cell lines (E2 and E4) by western blot and immunohistochemistry (Figure 4B and 4C). Interestingly, E cell lines showed slightly higher levels of *AR* expression than CE cell lines, suggesting that E cell lines may have acquired ADI growth though AR overexpression (Figure 4B). The ADI cell growth of CE cells (CE1, CE2, and CE3) was more notably impacted than E cells (E2 and E4) when treated with increasing levels of a TPL2 inhibitor (Figure 4D). Collectively, these observations suggest that *TPL2* is also required for efficient ADI prostate cancer cell growth in the mouse model.

### TPL2 expression is upregulated in human ADI prostate cancer specimens

**Figure 5:**
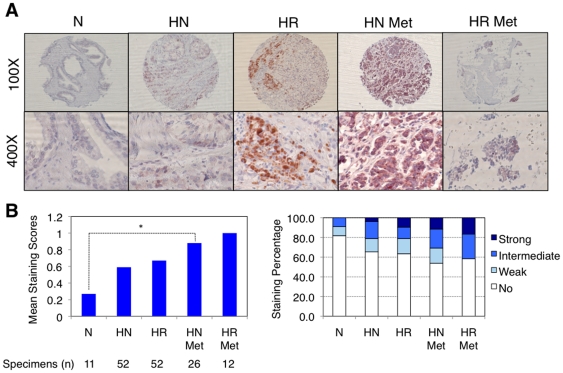
*TPL2* upregulation in ADI human prostate cancer specimens. (A) Representative photos of each normal prostate (N), hormone-naïve prostate cancer (HN), hormone-refractory prostate cancer (HR), hormone-naïve metastatic prostate cancer (HN Met), and hormone-refractory metastatic prostate cancer (HR Met) of the stained human prostate tissue microarrays. TPL2 expression was detected by immunohistochemical analysis using an antibody against TPL2. (B) Statistical analysis of TPL2 expression in each group of N, HN, HR, HN Met, HR Met samples of the stained human prostate tissue microarrays in terms of mean staining scores and staining percentage. Statistically significant differences in mean staining score is marked with * (p<0.05). Total sample numbers for each group are noted. Staining intensity was scored independently by two urologic pathologists with 0 (no staining), 1 (weak staining), 2 (intermediate staining), and 3 (strong staining) for each sample.

Finally, to validate the clinical relevance of *TPL2* kinase action in human ADI prostate cancer growth, we examined the frequency of *TPL2* expression in ADI human prostate cancer specimens using tissue microarrays (TMA). We evaluated the TPL2 immunostaining of 12 normal (N), 52 hormone-naïve (HN), 52 hormone-refractory (HR), 26 hormone-naïve metastatic (HN Met), and 12 hormone-refractory metastatic (HR Met) formalin fixed paraffin embedded samples. Figure 5A shows representative photos of the TPL2 expression of normal prostate, hormone-naive prostate cancer, and hormone-refractory prostate cancer human prostate TMA samples. As shown in Figure 5B, the overall staining frequency of TPL2 increased in hormone-naïve (34.8%) and hormone-refractory (36.5%) samples in comparison to normal prostate samples (18.2%). The increases are more significant in metastatic prostate cancer samples with 46.2% (p<0.05) of hormone-naïve metastatic and 41.7% of hormone-refractory metastatic samples, suggesting that TPL2 expression may also be associated with prostate cancer metastasis. In particular, both the overall mean staining score and the frequency of samples with a strong straining score increased with the progression of prostate cancer (Figure 5B). Taken together, these TMA data suggest that TPL2 expression is upregulated with the progression of prostate cancer.

## Discussion

Prostate cancer can easily be treated and cured if it is detected in its early stages, when tumor growth is still confined to the prostate. However, once the cancer spreads beyond the prostate gland, treatment becomes difficult, although treatment options, such as radiation therapy, hormone therapy, and chemotherapy are used with varying degrees of effectiveness. The emergence of a more aggressive, androgen depletion-independent (ADI) form of prostate cancer is complication frequent occurrence and reason for failure of hormone therapy. Therefore, studies associated with the identification and characterization of the genetics underlying ADI prostate cancer growth are critical for future developments of target-driven therapeutic strategies for the efficient treatment of ADI prostate cancer. In this study, by screening a library of activated human kinases, we identified and characterized a kinase gene, *TPL2*, that appears to drive ADI prostate cancer growth. More importantly, *TPL2* is upregulated in ADI prostate cancers of both the *Pten* deletion mouse model and the clinical prostate cancer specimens. These data strongly suggest that *TPL2* kinase plays a critical role in the promotion of ADI prostate cancer progression.

*TPL2*, also known as tumor progression locus 2, encoding a product with a deletion of its C-terminal region was initially cloned as a transforming gene from a human thyroid carcinoma cell line, and it was called *cot* (cancer Osaka thyroid) [35]. Almost at the same time, it was also identified as a protein kinase associated with the development of T-cell lymphoma, when it was discovered that the Moloney murine leukemia proviral genome had integrated into the last intron of the *TPL2* gene resulting in a deletion of its C-terminal [36]. A transgenic mouse study showed that only transgenic mice expressing a C-terminal deleted form of *TPL2* under the control of a proximal *Lck* promoter developed T-cell lymphoma, suggesting that the C-terminal domain of wild-type *TPL2* plays an inhibitory role in its kinase activity [37]. In fact, the C-terminal deleted TPL2 has increased stability and a higher specific kinase activity than wild-type. In addition, TPL2 interacts with a negative regulator, the NF-κB-inhibitory protein NF-κB1 p105 through its C-terminus, and thus the C-terminal deleted TPL2 is insensitive to p105 negative regulation [38], [39]. Interestingly, *TPL2* expression is also upregulated in approximately 40% of human breast cancer specimens, which may be caused by an increase in *TPL2* gene copy numbers [40]. Therefore, it is important to determine whether TPL2 signaling activation in human prostate cancer samples may be due to either the genetic mutation at a C-terminal region or a gene copy number increase. Our data showed that TPL2 is expressed at higher level in ADI-C4-2B cells than in AD-LNCaP cells, thereby contributing to the high endogenous level of ADI transcriptional activation of the *PSA* enhancer/promoter *cis*-regulatory region in the ADI-C4-2B cells (Figure S1). We also compared the *TPL2* sequences of two cell lines, but did not detect any sequence difference, suggesting that the ADI phenotype of C4-2B cells is not associated with specific mutations at the *TPL2* gene. In addition, a previous study showed that the mRNA level of *TPL2* in the mouse prostate increased after surgical castration, but it subsequently decreased after re-administration of testosterone [41]. These microarray data suggest that *TPL2* expression in the prostate is inversely regulated by testosterone at the transcriptional level. Therefore, it is possible that decreased level of androgen with hormone therapy may increase TPL2 expression, resulting in prostate cancer cell survival and proliferation under androgen-depleted conditions. This hypothesis is under an active investigation.

In the presence of androgen, AR undergoes phosphorylation, dimerization, and translocation into the nucleus, wherein it binds to ARE sites, resulting in the transcriptional activation of target genes [8]. However, several reports indicate that AR is still necessary for ADI prostate cancer growth [9], [10]. As for the underlying mechanisms of TPL2-mediated ADI prostate cancer growth, a question is whether this action requires AR expression and activity. The abrogation of TPL2-mediated PSA promoter activation in AR-negative DU-145 prostate cancer cells suggests that *TPL2*-mediated ADI prostate cancer growth may be still dependent on AR activity. However, this notion may be more complex since because DU-145 cells also lack co-regulatory factors associated with the AR signaling [42]. To determine whether AR is required for TPL2-mediated transcriptional activation of the *PSA* enhancer/promoter *cis*-regulatory region under androgen-depleted conditions, we suppressed *AR* expression by shRNA-mediated knockdown. Importantly, as shown in Figure S5, the decreased expression of *AR* is correlated with decreasing TPL2-mediated ADI transcriptional activation of the *PSA* enhancer/promoter *cis*-regulatory region. Therefore, TPL2-mediated ADI transcriptional activation of the *PSA* enhancer/promoter *cis*-regulatory region is AR-dependent. These data suggest TPL2 activation induces AR function under androgen-depleted conditions, possibly through post-translational modifications, such as phosphorylation. Activated TPL2 kinase itself or its downstream kinases may phosphorylate AR, which induces the dimerization and nuclear translocation of AR, and ultimately activates its target gene expressions for ADI prostate cancer cell growth. On the other hand, it would also be interesting to investigate whether TPL2-mediated ADI prostate growth is due to the epigenetic modulation of androgen-responsive genes by TPL2 in the absence of androgen, as TPL2 phosphorylates the histone H3 to induce epigenetic alteration and thereby cell growth transformation [43], [44].

Chronic prostatic inflammation has been proposed to be a major factor influencing prostate cancer, although the etiological agents of prostatic inflammation remain controversial [45]. Recently, genetically-engineered mouse studies clearly shows that an inflammatory cytokine, a lymphotoxin produced by B cells which have infiltrated the site of regressing AD prostate cancer after castration, promotes ADI prostate cancer [46]. It has also been shown that Toll-like receptor 4 (TLR4) is expressed in the prostate gland and its expression is closely associated with the severity of prostate cancer [47]. Therefore, these data support a connection between the activation of the TPL2 signaling pathway and inflammatory responses in the development prostate cancer. Furthermore, epidemiological studies have suggested that exposure to inorganic arsenite is an important environmental risk factor of human prostate cancer [48]. Importantly, TPL2 has been identified as a key regulator of arsenite-induced signal transduction [49]. Thus, future study will be directed to investigate the role of TPL2-mediated inflammatory signal transduction in ADI prostate cancer development.

Functionally, *TPL2* activation induces ERK, JNK, and NF-κB signaling pathways in both stimulus- and cell type-specific manners [38], [50]. The treatment of a MEK inhibitor, U0126, significantly diminished TPL2-mediated transcriptional activity of the *PSA* enhancer/promoter *cis*-regulatory region reporter construct under androgen-depleted conditions (Figure S6). In addition, either overexpression of a dominant-negative mutant form of I**κ**Bα, I**κ**Bα (32/36AA) denoted as I**κ**Bα DN, or treatment of a IKK inhibitor, PS-1145, diminished the PSA transcriptional activity (Figure S7). Together, these data suggest that the activation of both the MEK/ERK pathway and NF-κB signaling pathway is required for TPL2-mediated ADI prostate cancer growth. Therefore, we propose that a combination targeting both the TPL2/MEK/ERK pathway and the RAS/RAF/MEK/ERK pathway may potentially lead to new therapeutic intervention strategies of ADI prostate cancer (Figure S8).

## Materials and Methods

### Screening of a Library of Activated Human Kinases

The library of 354 human kinases contains 98 kinase-related open reading frames (ORFs) and 256 human kinases cloned into a Gateway-compatible retroviral destination vector, pWN-MF-DEST, in which a myr sequence and a flag-epitope tag (MF) were added to the N-termini of each introduced ORF [27]. 1 µg DNA of each kinase construct, along with 0.5 µg DNA of a reporter plasmid containing the 5.8 kb enhancer/promoter region of the PSA gene (PSA-enhancer/promoter-Luc), were co-transfected into individual wells of a 12-well plate containing 2×10^5^ androgen-dependent LNCaP (AD-LNCaP) cells using Lipofectamine™ LTX (3 µl of Lipofectamine LTX+1 µl of *plus* reagent in 100 µl opti-MEM for each transfection; Invitrogen). 24 hours after transfection, the media was changed with 10% charcoal-stripped FBS (CS-FBS) with/without R1881, a synthetic androgen. Relative LUC activity (the PSA-enhancer/promoter-luciferase activity normalized to the pGL3-beta-galactosidase activity) was measured after 24 hours of further incubation using Dual-Luciferase® Reporter Assay System and beta-galactosidase Enzyme Assay System (Promega). All the transfections were performed independently twice.

### Cell Lines, Vectors, and Retroviral/Lentiviral Infection

293T cells and LNCaP cells were obtained from ATCC (Manassas, VA). C-2B cells, previously established from LNCaP cells [51], were kindly provided by Dr. Roy-Burman. We first established AD-LNCaP stable cell lines that over-express either a N-terminally myc-tagged wild-type [*myc*-*TPL2* (WT)], a N-terminally myc-tagged constitutively activated form with a deletion of 70 amino acids in C-terminus [*myc*-*TPL2* (ΔC)], or a N-terminally myc-tagged kinase-inactive form of *TPL2* [*myc*-*TPL2* (D270A)] using a pBabe-puro retroviral expression vector. 293T cells (4×10^6^ cells per 10 Cm dish) were transfected with 5 µg of each expression vector and 5 µg of packaging vector (pCL) using the CalPhos™ Mammalian Transfection Kit (Clontech). After 6 hours, the media was changed, and the medium containing the recombinant viruses was collected at 48 and 72 hours post transfection for two independent infections. For each infection, the collected media was filtered through a 0.45 µm filter to remove cell debris and then equal amounts of fresh RPMI supplemented with 10% FBS was mixed with the filtered media with 8 µl of polybrene (1 mg/L) per 10 ml medium. 24 hours after the second infection, infected cells were selected for one week with puromycin (1 µg/ml). The established cell lines were then checked for *TPL2* expression by western blot using an antibody against TPL2. Another set of AD-LNCaP stable cell lines that over-express either a C-terminally flag-tagged wild-type (*TPL2*-wt-flag), a C-terminally flag-tagged constitutively activated form with a deletion of 70 amino acids in C-terminus (*TPL2*-delC-flag), or a C-terminally flag-tagged kinase-inactive form of *TPL2* (*TPL2*-inactive-flag) were established using a pEF-IRES-puro expression vector. Each construct (5 µg) was transiently transfected into LNCaP cells using the Lipofectamine™ LTX with Plus™ Reagent (Invitrogen), and transfected cells were selected for one month with puromycin (1 µg/ml). For shRNA-mediated knockdown, we established two sets of ADI-C4-2B stable cell lines that express either shRNA-control or shRNA-*TPL2* using both the pGIPZ lentiviral vector and the pLKO.1-TRC lentiviral expression vector. In brief, 7.5 µg of pCMV-dR8.2 dvpr (Addgene) and 3 µg of pCMV-VSV-G (Addgene) packaging vectors were used with 10 µg of lentiviral expression vector for transfection into 293T cells. We used three different antibodies to detect TPL2. An antibody that detects an N-terminal epitope of TPL2 (Cot [N-17], catalog # sc-1717, Santa Cruz INC. with 1∶500 dilution) was used for the detection of TPL2 expression by western blot in Figure 2B, Figure 4B, Figure S2B, and Figure S3A. An antibody that detects an C-terminal epitope of TPL2 (Cot [M-20], catalog # sc-720, Santa Cruz INC. with 1∶500 dilution) was also used for the detection of TPL2 expression by western blot in Figure 3A and Figure S1. An antibody that detects phosphorylated Threonine at the 290 site of TPL2 (MAP3K8 [phospho T290], catalog # ab51214, abcam® with 1∶200 dilution) was used by western blot in Figure S2B. Also, we purchased a TPL2 inhibitor and a MEK inhibitor (U0126) from Calbiochem (Cat. No. 616373) and Cell Signaling Technology (Cat. No. 9903), respectively.

### MTT Assay

The number of viable cells in proliferation was measured according to the manufacture's protocol using the CellTiter 96® AQ_ueous_ One Solution Cell Proliferation Assay (Promega). 5,000 cells in a volume of 200 µl were seeded in an individual well of 96-well plates.

### Immunohistochemical Analysis

TMAs composed of 11 cases of normal prostate, 52 cases of hormone-naïve human prostate cancer tissue, 52 cases of hormone-refractory prostate cancer tissue, 26 cases of hormone-naïve metastatic prostate cancer tissue, and 12 cases of hormone-refractory metastatic prostate cancer tissue were obtained from the New York University (NYU) Prostate Cancer Tissue Resource. The TMAs were incubated in pre-boiled citrate buffer (0.01 M, PH 6.4) and left until they reached room temperature (R.T.) for antigen retrieval, and immunostained with a TPL2 antibody (Cot (M-20) #sc-720; Santa Cruz Biotechnology Inc.; 1∶200 dilution). As a positive control, human duodenum tissue samples were used to optimize staining conditions. The staining was reviewed and expression of *TPL2* scored for intensity and proportion by Dr. Jonathan Melamed at NYU.

## Supporting Information

Figure S1 **Higher level of TPL2 expression in ADI-C4-2B cells than AD-LNCaP cells.** (A) Western blot analysis was performed to compare the level of TPL2 expression between AD-LNCaP cells and ADI-C4-2B cells (three different passages for each cell line). *β-actin* was used as a control for loading the same amount of proteins. (B) The PSA enhancer/promoter activity in response to overexpression of exogenous TPL2 in ADI-C4-2B cells. Relative LUC activities were measured with increasing concentration of TPL2 expression vector in ADI-C4-2B cells. All the experiments were performed three times with each in triplicate, and each column represents the mean ± standard deviation.
(TIF)

Figure S2 **Induction of ADI prostate cancer cell growth by *TPL2* activation.** (A) Schematic of established stable LNCaP cell lines overexpressing either a C-terminally flag-tagged wild-type *TPL2* (*TPL2*-wt-flag), a C-terminally flag-tagged constitutively activated form of *TPL2* with a truncation of 70 amino acids in its C-terminus (*TPL2*-delC-flag), or a C-terminally flag-tagged kinase-inactive form of *TPL2* (*TPL2*-inactive-flag) using the pEF-IRES-puro expression vector. (B) Western blot analysis to detect the expression of TPL2 and its downstream signaling molecules. TPL2 (N-terminal), TPL2 (C-terminal), and TPL2 (phospho-270) expression detected by different antibodies that specifically recognize a N-terminal epitope, a C-terminal epitope, or phopsphorylation at Threonine 290 of TPL2 (as indicated with arrows in red), respectively. β-actin was used as a control for loading the same amount of proteins. (C) MTT assays to measure cell proliferation of the stable cell lines above in the absence of R1881. Increased cell proliferation of C4-2B (vector), LNCaP (TPL2-wt-flag), and LNCaP (TPL2-delC-flag) cells under androgen-depleted conditions at 2, 4, 6, and 8 days are all statistically significant in comparison to the cell proliferation of LNCaP (vector) cells (* p<0.05).
(TIF)

Figure S3 **Inhibition of ADI prostate cancer growth by *TPL2* suppression.** (A) Western blot analysis to show the levels of *TPL2* expression in the stable C4-2B cell lines expressing the indicated shRNAs using a pGIPZ lentiviral expression vector at the time of the following MTT assay. The number indicates different shRNA clones targeting the same gene. Because TPL2/MAP3K8 is a MAP3K (mitogen-activated protein kinase kinase kinase), two other MAP3Ks, MAP3K6 (mitogen-activated protein kinase kinase kinase 6) and MAP3K14 (mitogen-activated protein kinase kinase kinase 14) were used as controls for off-target effects. β-actin was used as a control for loading the same amount of proteins. (B) MTT assays to measure cell proliferation of C4-2B stable cell lines expressing either shRNA (#4) targeting TPL2, which showed efficient knockdown of TPL2 expression above, or control shRNA under androgen-depleted conditions. Cell proliferation of C4-2B stable cells expressing shRNA (#4) targeting TPL2 significantly decreased in comparison to control cells on the indicated days (* p<0.05).
(TIF)

Figure S4 **Determination of the 50% inhibitory concentration (IC^50^) of a TPL2 inhibitor.** MTT assays measuring the cell proliferation of ADI-C4-2B cells in the absence of R1881 with increasing concentration of the TPL2 inhibitor were performed to determine the 50% inhibitory concentration (IC^50^) of a TP2 inhibitor. After 24 hours of TPL2 inhibitor treatment at a concentration of 4 uM, cell proliferation of ADI-C4-2B cells was suppressed by 50% in comparison to no treatment. The same concentrations of vehicle (DMSO) were treated in parallel as controls.
(TIF)

Figure S5 **TPL2-mediated ADI transcriptional activation of the *PSA* enhancer/promoter *cis*-regulatory region is AR-dependent.** Relative LUC activities were measured under androgen-depleted conditions with increasing amount of sh-RNAs targeting AR. AD-LNCaP cells were transiently co-transfected with a reporter plasmid (the *PSA* enhancer/promoter *cis*-regulatory region reporter construct), a plasmid expressing a constitutively activated form of *TPL2* (myristoylated TPL2), and the indicated sh-RNAs. Western blot analysis shows the amount of *AR* expression at the time of the Luciferase assay. β-actin was used as a control for loading the same amount of proteins. Statistically significant decreases in LUC activities in comparison to that of TPL2-mediated ADI trans-criptional activation (0 nM R1881+TPL2) are marked with * (p<0.05).
(TIF)

Figure S6 **The activation of MEK/ERK pathway is required for TPL2-mediated ADI prostate cancer growth.** (A) Relative LUC activities were measured with increasing concentration of a MEK inhibitor (U0126) in AD-LNCaP cells that were transiently co-transfected with a reporter plasmid (the *PSA* enhancer/promoter *cis*-regulatory region reporter construct) and a plasmid expressing a constitutively activated form of *TPL2* (myristoylated TPL2). Relative LUC activities with the transfection of a vector control with/without R1881 are indicated in black bars. All the experiments were performed three times with each in triplicate, and each column represents the mean ± standard deviation. Statistically significant decreases in LUC activities in comparison to vehicle treatment are marked with * (p<0.05). (B) Western blot analysis shows the inhibition of ERK/MEK pathway in LNCaP cells with increasing amount of U0126 treatment.
(TIF)

Figure S7 **The activation of NF-κB pathway is also required for TPL2-mediated ADI prostate cancer growth.** (A) and (B) Relative LUC activities were measured with increasing concentration of either a dominant-negative mutant form of I**κ**Bα, I**κ**Bα (32/36AA) deonoted as I**κ**Bα DN, or a IKK inhibitor, PS-1145. In AD-LNCaP cells, reporter plasmids (NF-κB promoter-Luc or *PSA* enhancer/promoter-Luc) were transiently co-transfected with either a plasmid expressing a constitutively activated form of *TPL2* (myristoylated TPL2) or control vector. Statistically significant decreases in LUC activities with increasing amount of I**κ**Bα DN in comparison to vector control with TPL2 expression (the third column) are marked with * (p<0.05). In ADI-C4-2B cells, only the reporter plasmids were transfected. All the experiments were performed three times with each in triplicate, and each column represents the mean ± standard deviation. Statistically significant decreases in LUC activities with increasing amount of I**κ**Bα DN in comparison to vehicle treatment are marked with * (p<0.05). (C) MTT assays measuring the cell proliferation of indicated cells in the absence of R1881 with the treatment of PS-1145 (10 uM) were performed. Statistically significant decreases in cell proliferation in comparison to mock treatment are marked with * (p<0.05).
(TIF)

Figure S8 **Alternative activation of the MEK/ERK pathway and NF-κB by TPL2 contribute to ADI prostate cancer cell growth.** Previously, it has been shown that activation of AKT and ERK MAP kinase signaling pathways synergistically promote *in vivo* ADI prostate cancer growth, possibly through the activation of growth factors/receptor tyrosine kinase (RTK)/RAS pathway by autocrine and paracrine growth factor loops [38]. Here, we found TPL2 contribute to ADI prostate cancer growth through the alternative activation of MEK/ERK pathway and NF-κB. Therefore, our findings may potentially lead to new therapeutic intervention strategies for the treatment of ADI prostate cancer, such as a combination therapy targeting both TPL2 activation and the RAS/RAF/MEK/ERK pathway for example. RTK, receptor tyrosine kinase; SHC, Src homology 2 domain containing transforming protein; GRB 2, growth factor receptor bound 2; GEF, guanine nucleotide exchange factor; GAP, GTPase activating protein.
(TIF)
