# Supplementary figures and images for: TPL2/COT/MAP3K8 (TPL2) Activation Promotes Androgen Depletion-Independent (ADI) Prostate Cancer Growth
Source: PLoS One. 2011 Jan 18;6(1):e16205. doi: 10.1371/journal.pone.0016205 (PMC3022761; doi:10.1371/journal.pone.0016205)

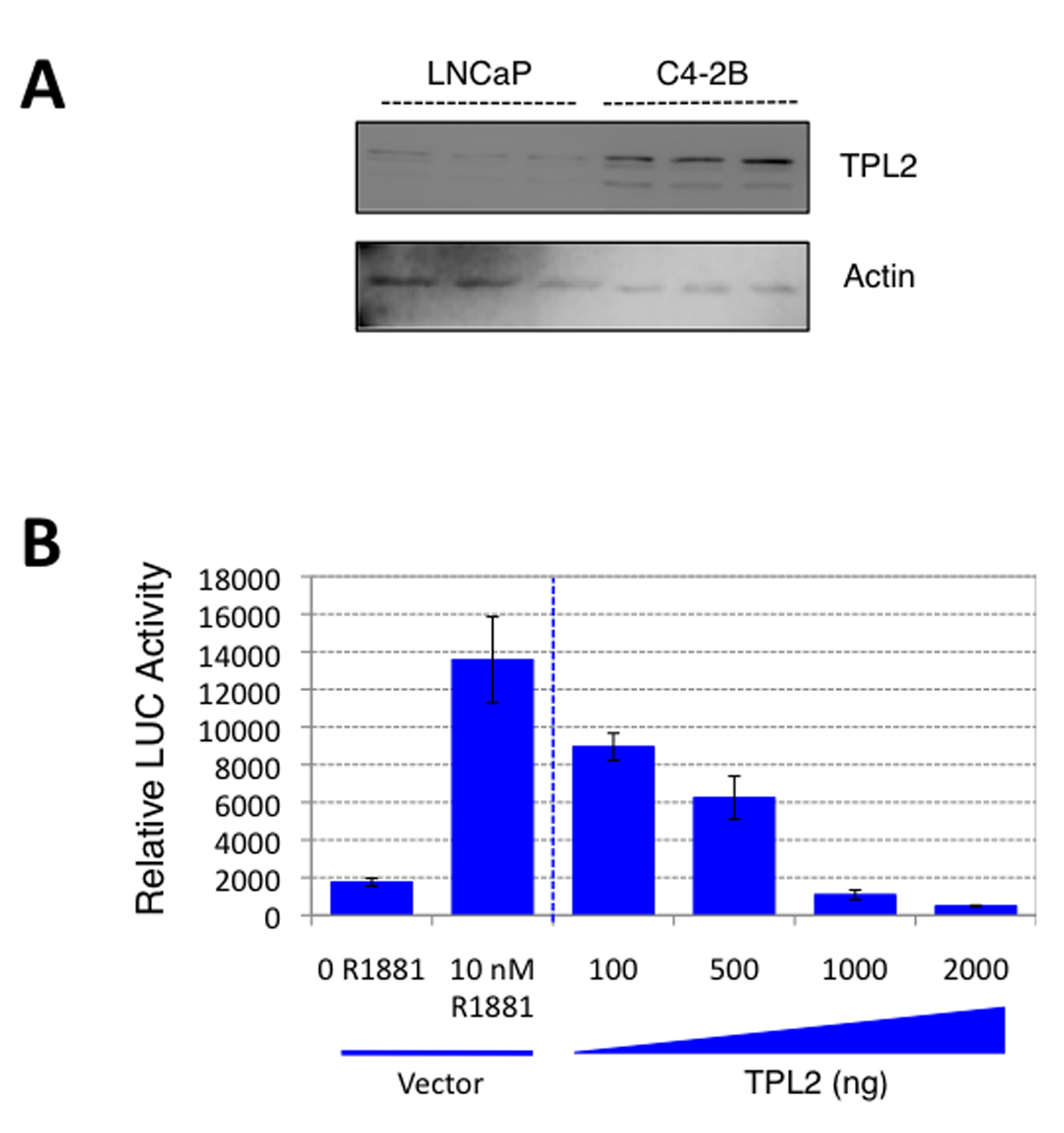

Supplement: Figure S1 — Higher level of TPL2 expression in ADI-C4-2B cells than AD-LNCaP cells. (A) Western blot analysis was performed to compare the level of TPL2 expression between AD-LNCaP cells and ADI-C4-2B cells (three different passages for each cell line). β-actin was used as a control for loading the same amount of proteins. (B) The PSA enhancer/promoter activity in response to overexpression of exogenous TPL2 in ADI-C4-2B cells. Relative LUC activities were measured with increasing concentration of TPL2 expression vector in ADI-C4-2B cells. All the experiments were performed three times with each in triplicate, and each column represents the mean ± standard deviation. (TIF) [file pone.0016205.s001.tif]

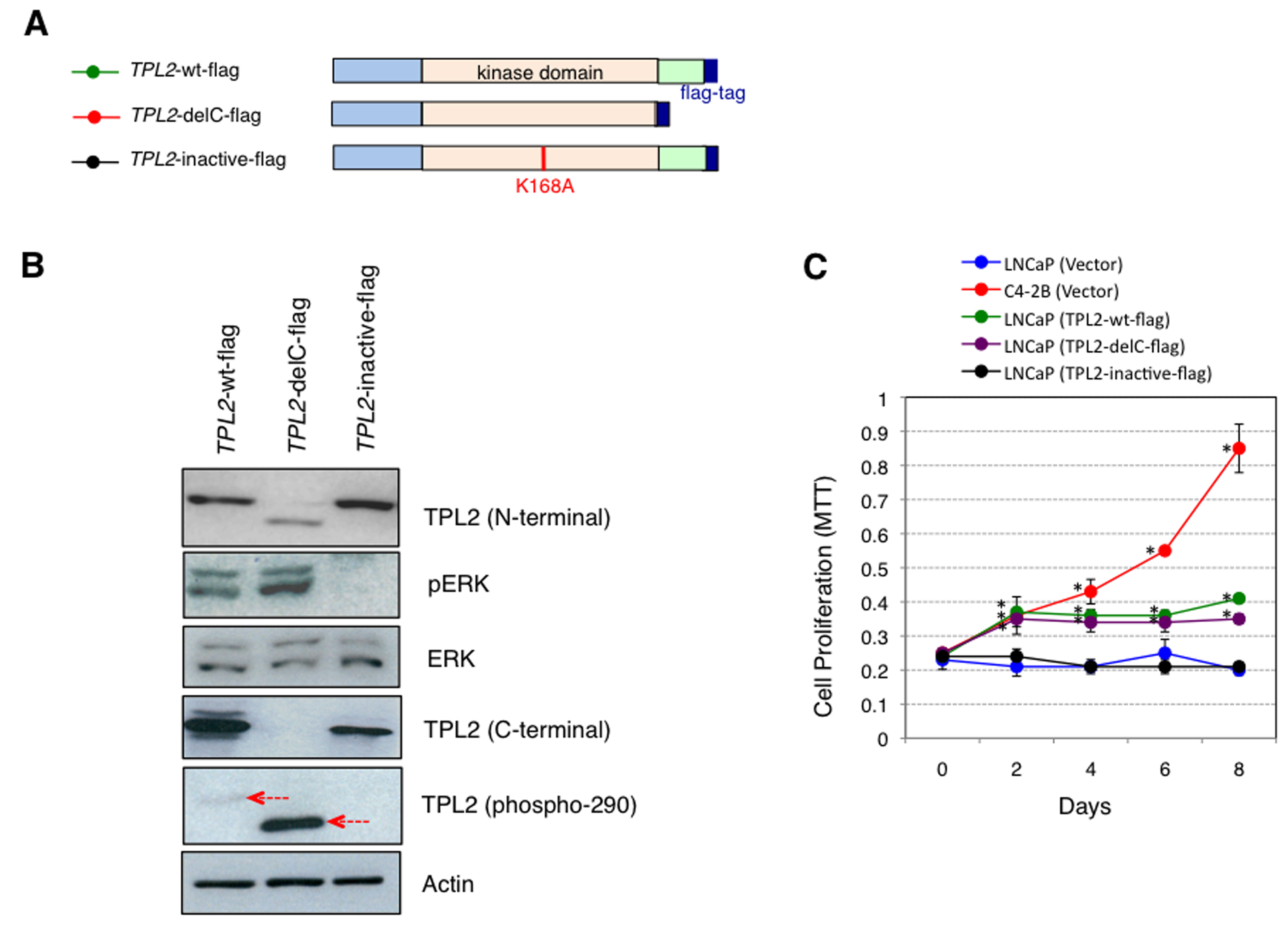

Supplement: Figure S2 — Induction of ADI prostate cancer cell growth by TPL2 activation. (A) Schematic of established stable LNCaP cell lines overexpressing either a C-terminally flag-tagged wild-type TPL2 (TPL2-wt-flag), a C-terminally flag-tagged constitutively activated form of TPL2 with a truncation of 70 amino acids in its C-terminus (TPL2-delC-flag), or a C-terminally flag-tagged kinase-inactive form of TPL2 (TPL2-inactive-flag) using the pEF-IRES-puro expression vector. (B) Western blot analysis to detect the expression of TPL2 and its downstream signaling molecules. TPL2 (N-terminal), TPL2 (C-terminal), and TPL2 (phospho-270) expression detected by different antibodies that specifically recognize a N-terminal epitope, a C-terminal epitope, or phopsphorylation at Threonine 290 of TPL2 (as indicated with arrows in red), respectively. β-actin was used as a control for loading the same amount of proteins. (C) MTT assays to measure cell proliferation of the stable cell lines above in the absence of R1881. Increased cell proliferation of C4-2B (vector), LNCaP (TPL2-wt-flag), and LNCaP (TPL2-delC-flag) cells under androgen-depleted conditions at 2, 4, 6, and 8 days are all statistically significant in comparison to the cell proliferation of LNCaP (vector) cells (* p<0.05). (TIF) [file pone.0016205.s002.tif]

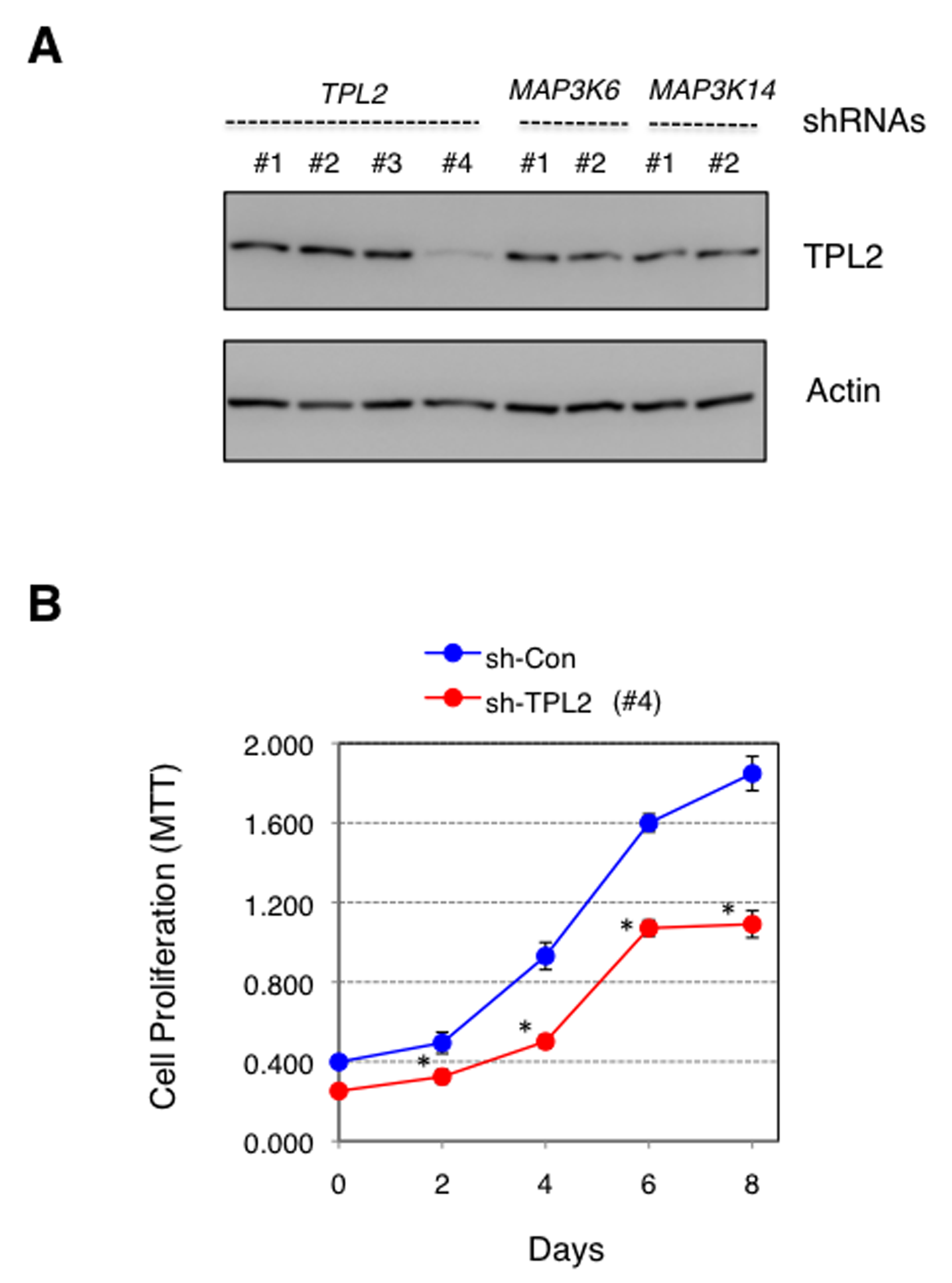

Supplement: Figure S3 — Inhibition of ADI prostate cancer growth by TPL2 suppression. (A) Western blot analysis to show the levels of TPL2 expression in the stable C4-2B cell lines expressing the indicated shRNAs using a pGIPZ lentiviral expression vector at the time of the following MTT assay. The number indicates different shRNA clones targeting the same gene. Because TPL2/MAP3K8 is a MAP3K (mitogen-activated protein kinase kinase kinase), two other MAP3Ks, MAP3K6 (mitogen-activated protein kinase kinase kinase 6) and MAP3K14 (mitogen-activated protein kinase kinase kinase 14) were used as controls for off-target effects. β-actin was used as a control for loading the same amount of proteins. (B) MTT assays to measure cell proliferation of C4-2B stable cell lines expressing either shRNA (#4) targeting TPL2, which showed efficient knockdown of TPL2 expression above, or control shRNA under androgen-depleted conditions. Cell proliferation of C4-2B stable cells expressing shRNA (#4) targeting TPL2 significantly decreased in comparison to control cells on the indicated days (* p<0.05). (TIF) [file pone.0016205.s003.tif]

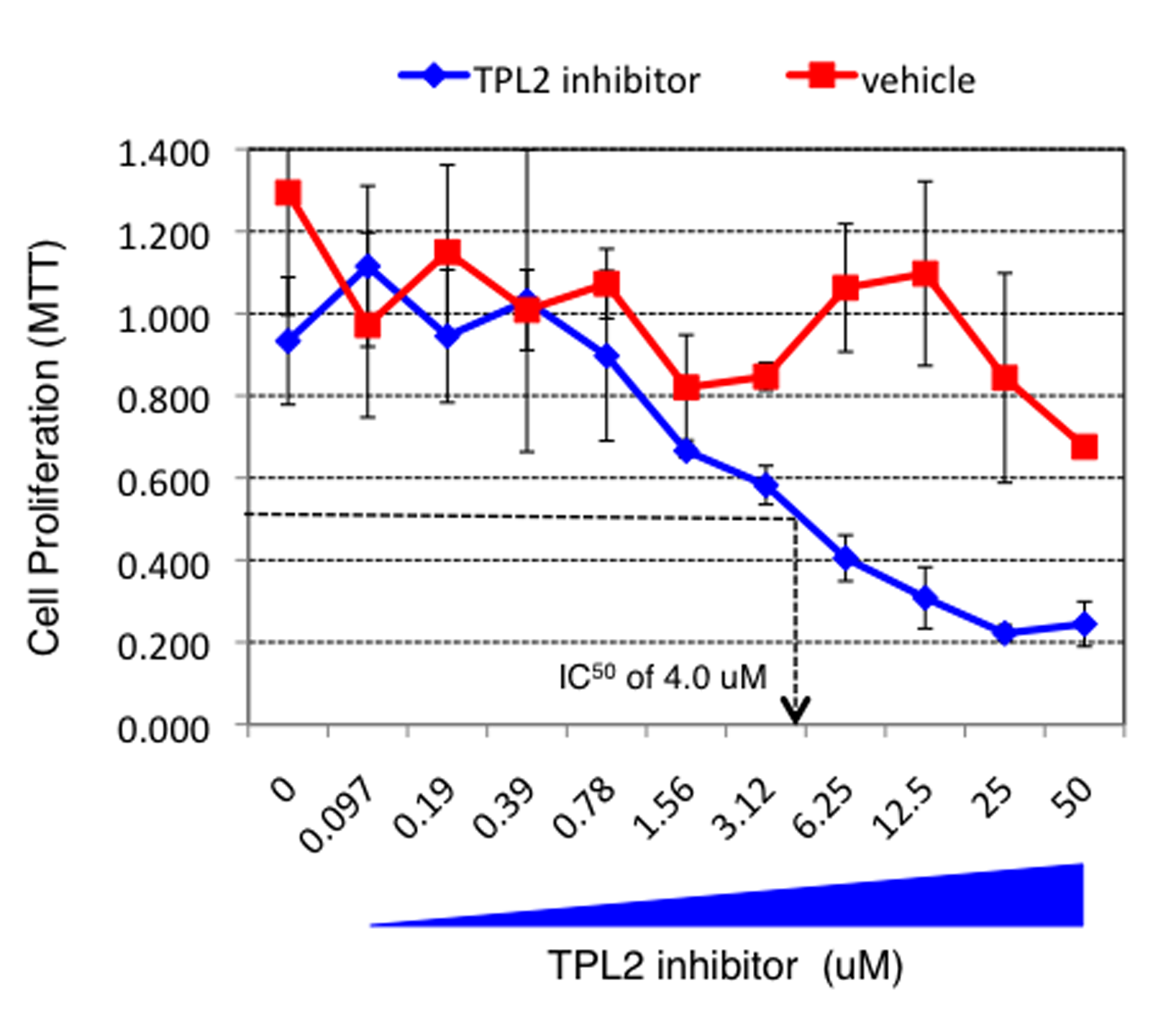

Supplement: Figure S4 — Determination of the 50% inhibitory concentration (IC50) of a TPL2 inhibitor. MTT assays measuring the cell proliferation of ADI-C4-2B cells in the absence of R1881 with increasing concentration of the TPL2 inhibitor were performed to determine the 50% inhibitory concentration (IC50) of a TP2 inhibitor. After 24 hours of TPL2 inhibitor treatment at a concentration of 4 uM, cell proliferation of ADI-C4-2B cells was suppressed by 50% in comparison to no treatment. The same concentrations of vehicle (DMSO) were treated in parallel as controls. (TIF) [file pone.0016205.s004.tif]

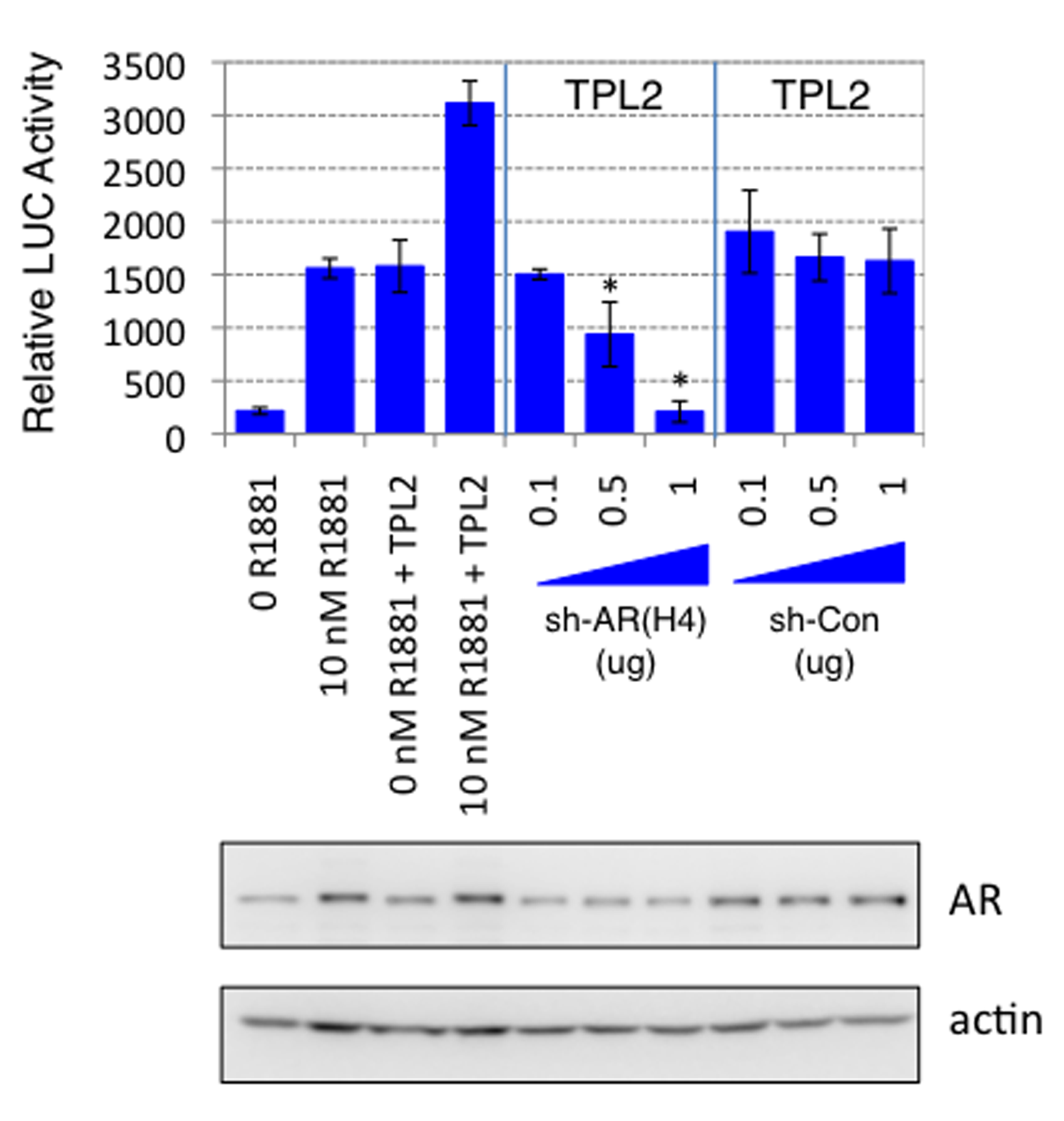

Supplement: Figure S5 — TPL2-mediated ADI transcriptional activation of the PSA enhancer/promoter cis-regulatory region is AR-dependent. Relative LUC activities were measured under androgen-depleted conditions with increasing amount of sh-RNAs targeting AR. AD-LNCaP cells were transiently co-transfected with a reporter plasmid (the PSA enhancer/promoter cis-regulatory region reporter construct), a plasmid expressing a constitutively activated form of TPL2 (myristoylated TPL2), and the indicated sh-RNAs. Western blot analysis shows the amount of AR expression at the time of the Luciferase assay. β-actin was used as a control for loading the same amount of proteins. Statistically significant decreases in LUC activities in comparison to that of TPL2-mediated ADI trans-criptional activation (0 nM R1881+TPL2) are marked with * (p<0.05). (TIF) [file pone.0016205.s005.tif]

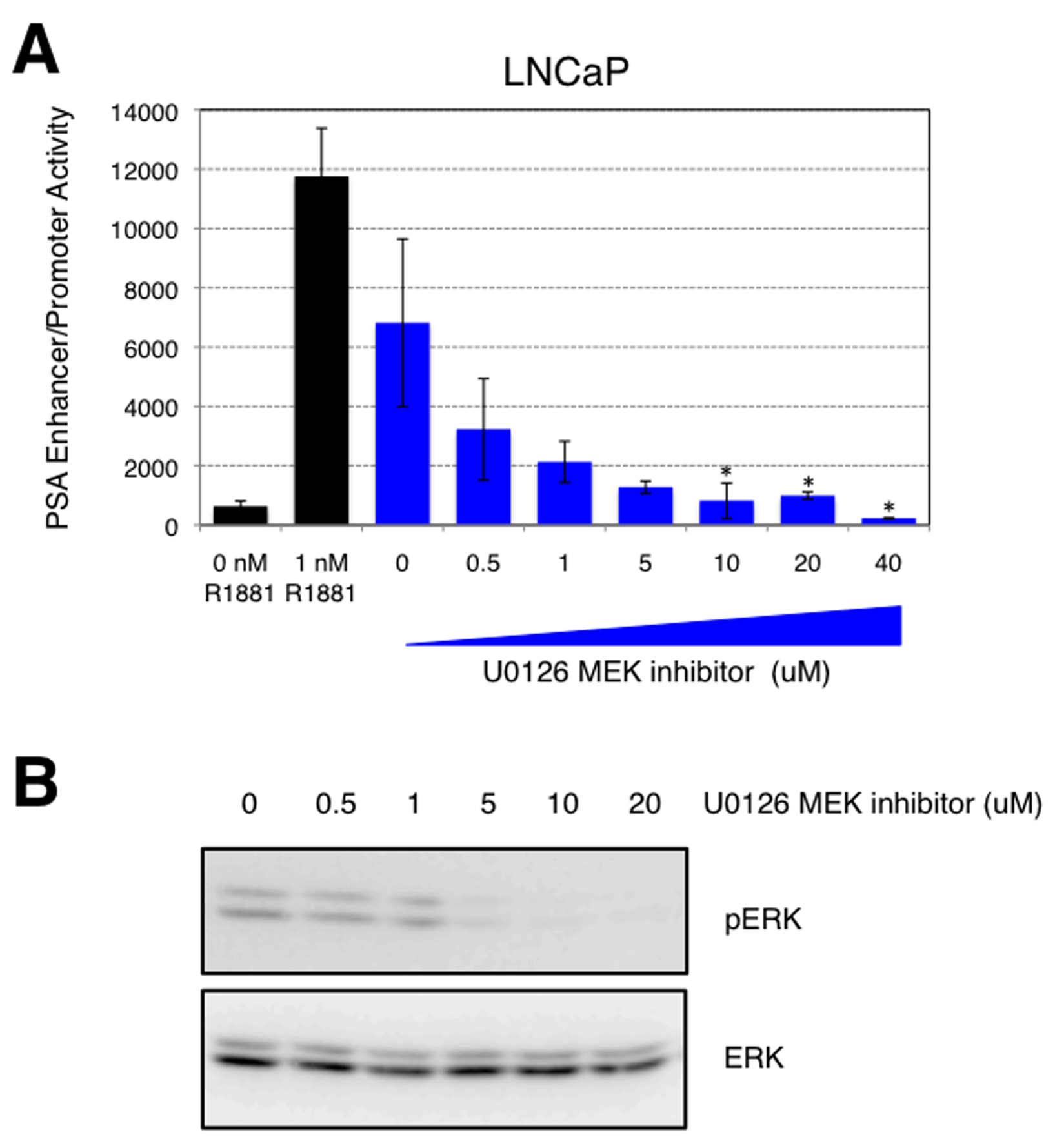

Supplement: Figure S6 — The activation of MEK/ERK pathway is required for TPL2-mediated ADI prostate cancer growth. (A) Relative LUC activities were measured with increasing concentration of a MEK inhibitor (U0126) in AD-LNCaP cells that were transiently co-transfected with a reporter plasmid (the PSA enhancer/promoter cis-regulatory region reporter construct) and a plasmid expressing a constitutively activated form of TPL2 (myristoylated TPL2). Relative LUC activities with the transfection of a vector control with/without R1881 are indicated in black bars. All the experiments were performed three times with each in triplicate, and each column represents the mean ± standard deviation. Statistically significant decreases in LUC activities in comparison to vehicle treatment are marked with * (p<0.05). (B) Western blot analysis shows the inhibition of ERK/MEK pathway in LNCaP cells with increasing amount of U0126 treatment. (TIF) [file pone.0016205.s006.tif]

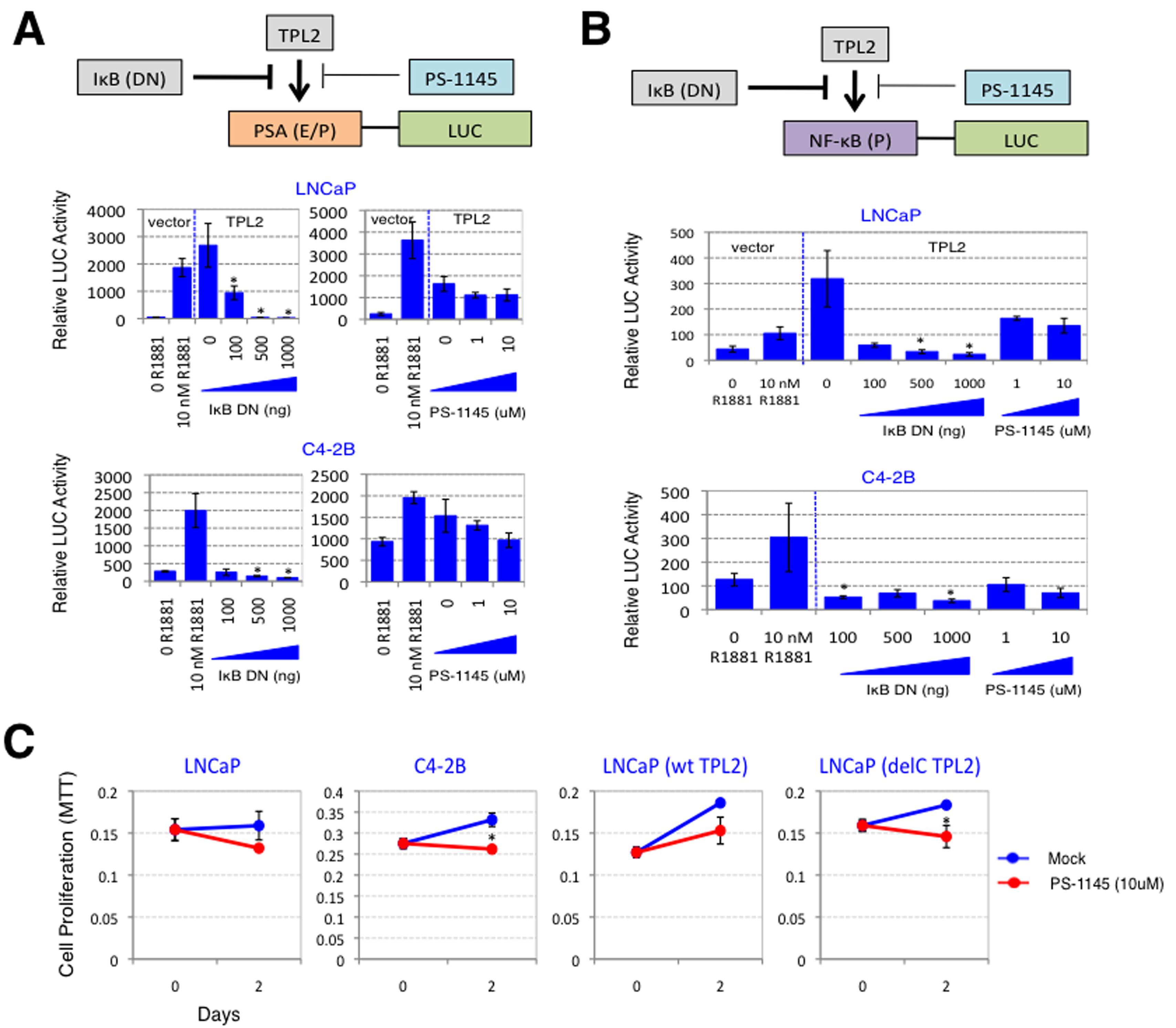

Supplement: Figure S7 — The activation of NF-κB pathway is also required for TPL2-mediated ADI prostate cancer growth. (A) and (B) Relative LUC activities were measured with increasing concentration of either a dominant-negative mutant form of IκBα, IκBα (32/36AA) deonoted as IκBα DN, or a IKK inhibitor, PS-1145. In AD-LNCaP cells, reporter plasmids (NF-κB promoter-Luc or PSA enhancer/promoter-Luc) were transiently co-transfected with either a plasmid expressing a constitutively activated form of TPL2 (myristoylated TPL2) or control vector. Statistically significant decreases in LUC activities with increasing amount of IκBα DN in comparison to vector control with TPL2 expression (the third column) are marked with * (p<0.05). In ADI-C4-2B cells, only the reporter plasmids were transfected. All the experiments were performed three times with each in triplicate, and each column represents the mean ± standard deviation. Statistically significant decreases in LUC activities with increasing amount of IκBα DN in comparison to vehicle treatment are marked with * (p<0.05). (C) MTT assays measuring the cell proliferation of indicated cells in the absence of R1881 with the treatment of PS-1145 (10 uM) were performed. Statistically significant decreases in cell proliferation in comparison to mock treatment are marked with * (p<0.05). (TIF) [file pone.0016205.s007.tif]

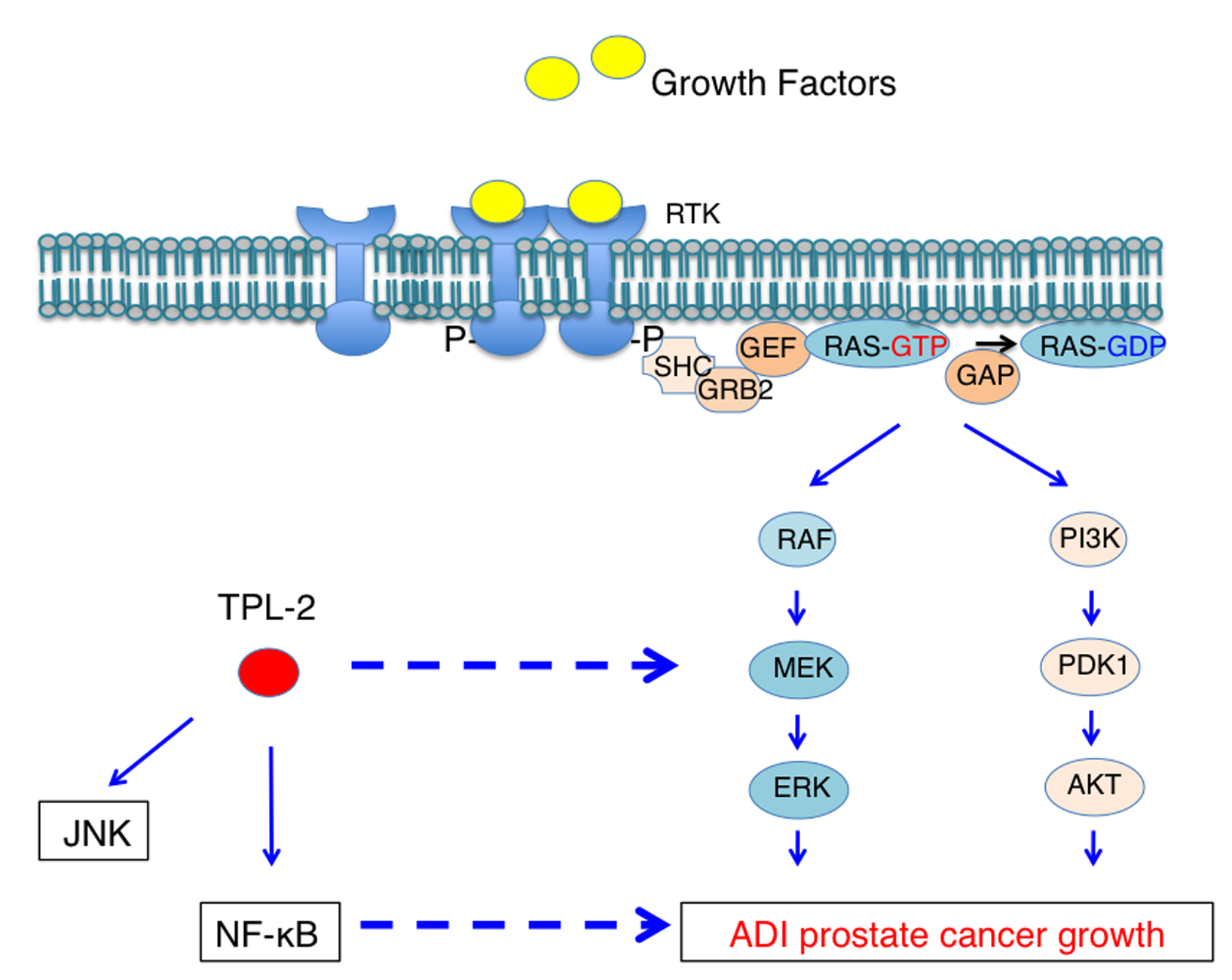

Supplement: Figure S8 — Alternative activation of the MEK/ERK pathway and NF-κB by TPL2 contribute to ADI prostate cancer cell growth. Previously, it has been shown that activation of AKT and ERK MAP kinase signaling pathways synergistically promote in vivo ADI prostate cancer growth, possibly through the activation of growth factors/receptor tyrosine kinase (RTK)/RAS pathway by autocrine and paracrine growth factor loops [38]. Here, we found TPL2 contribute to ADI prostate cancer growth through the alternative activation of MEK/ERK pathway and NF-κB. Therefore, our findings may potentially lead to new therapeutic intervention strategies for the treatment of ADI prostate cancer, such as a combination therapy targeting both TPL2 activation and the RAS/RAF/MEK/ERK pathway for example. RTK, receptor tyrosine kinase; SHC, Src homology 2 domain containing transforming protein; GRB 2, growth factor receptor bound 2; GEF, guanine nucleotide exchange factor; GAP, GTPase activating protein. (TIF) [file pone.0016205.s008.tif]
